# Human milk affects TLR4 activation and LPS-induced inflammatory cytokine expression in Caco-2 intestinal epithelial cells

**DOI:** 10.1038/s41598-024-64000-z

**Published:** 2024-06-11

**Authors:** Catherine R. Pizzarello, Ashley Nelson, Ilya Verekhman, Antti E. Seppo, Kirsi M. Järvinen

**Affiliations:** 1 Division of Pediatric Allergy and Immunology, Center for Food Allergy, Department of Pediatrics, University of Rochester School of Medicine and Dentistry, Golisano Children’s Hospital, 601 Elmwood Ave, Box 777, Rochester, NY 14642 USA; 2 Department of Microbiology and Immunology, University of Rochester School of Medicine and Dentistry, Rochester, NY USA

**Keywords:** Immunology, Intestinal epithelium, Innate immunity, TLR4, Albumin, Cytokines, Immunology, Cytokines, Innate immunity, Mucosal immunology, Neonatology, Paediatric research

## Abstract

Human milk (HM) components affect immune cell toll-like receptor 4 (TLR4) signaling. However, studies examining the immunomodulatory impacts of HM on TLR4 signaling in intestinal epithelial cells (IECs) are limited. This study utilized both a TLR4 reporter cell line and a Caco-2 IEC model to examine the effects of HM on lipopolysaccharide (LPS)-induced TLR4 activation and cytokine responses, respectively. Additionally, we performed fast protein liquid chromatography and mass spectrometry to identify a HM component that contributes to the effect of HM on LPS/TLR4 signaling. HM enhances LPS-induced TLR4 signaling as well as LPS-induced IEC gene expression of pro-inflammatory cytokines and negative regulators of NF-κB. Human serum albumin (HSA) present in HM contributes to these effects. HSA within HM synergizes with LPS to induce IEC gene expression of pro-inflammatory cytokines and negative regulators of NF-κB. Altogether, this study provides mechanistic evidence behind the immunomodulatory function of HM on IECs, which may contribute to an enhanced immune response in breast-fed neonates.

## Introduction

Human milk (HM) not only provides a source of nutrients for the infant, but also plays an essential role in the development of the infant mucosal immune system^1,2^. Breastfeeding has been associated with decreased risk of developing gastrointestinal infections in term infants as well as decreased risk of necrotizing enterocolitis (NEC) among preterm infants^3^. Several immunologically active components in HM such as immunoglobulin A (IgA), human milk oligosaccharides (HMOs), glycoproteins, and cytokines contribute to the protective properties of HM by altering gut microbiome composition and innate immune function^1^.

Recognition of microbe-associated molecular patterns (MAMPs) is a crucial part of proper innate immune function and the establishment of immune homeostasis. This process is largely mediated by pattern recognition receptors (PRRs) such as toll-like receptors (TLRs). Interestingly, components of HM, such as HMOs, soluble CD14 (sCD14), lactoferrin, and lactadherin have been shown to impact TLR signaling. Many of these factors possess anti-inflammatory properties by influencing the TLR4 pathway, suggesting that HM has the capacity to influence innate immune function and establishment of immune homeostasis^1^. Most studies on the immunomodulatory function of HM have focused on the effect of HM on professional immune cells, but only a handful of studies have examined the effect of HM on intestinal epithelial cells (IECs)^4,5^.

Through its passage in the gastrointestinal tract, HM encounters IECs prior to engagement with professional immune cells. There is growing evidence supporting IECs’ function not only as a barrier, but also as a coordinator of mucosal immunity^6^. IEC TLR signaling is one of the few pathways that regulate IEC immune function^7,8^. IECs are constitutively exposed to TLR ligands, such as the TLR4 ligand lipopolysaccharide (LPS). Although several HM components that individually affect TLR4 signaling have been identified^1^, our understanding of the basic mechanisms by which HM as a whole influences microbial sensing and subsequent immune responses in the infant gastrointestinal tract is lacking.

In the present study, we further explored the effects of HM on TLR4 activation using TLR4 transfected human embryonic kidney (HEK) cells (HEK-hTLR4) as well as the IEC colon cancer (Caco-2) cell line. Through these models, we demonstrate that HM enhances TLR4 activation by LPS as well as inflammatory cytokine and NF-κB negative regulator expression in Caco-2 cells. Finally, we establish that human serum albumin (HSA) in HM contributes to this enhancement. According to prior literature, HSA possesses the capacity to deliver LPS to its obligatory co-receptors sCD14^9^ and myeloid differentiation factor 2 (MD-2)^10^, facilitating activation of TLR4. Our study is the first to identify HSA as a component in HM that has immunomodulatory effects on IECs.

## Results

### Human milk enhances TLR4 activation by LPS

**Figure 1:**
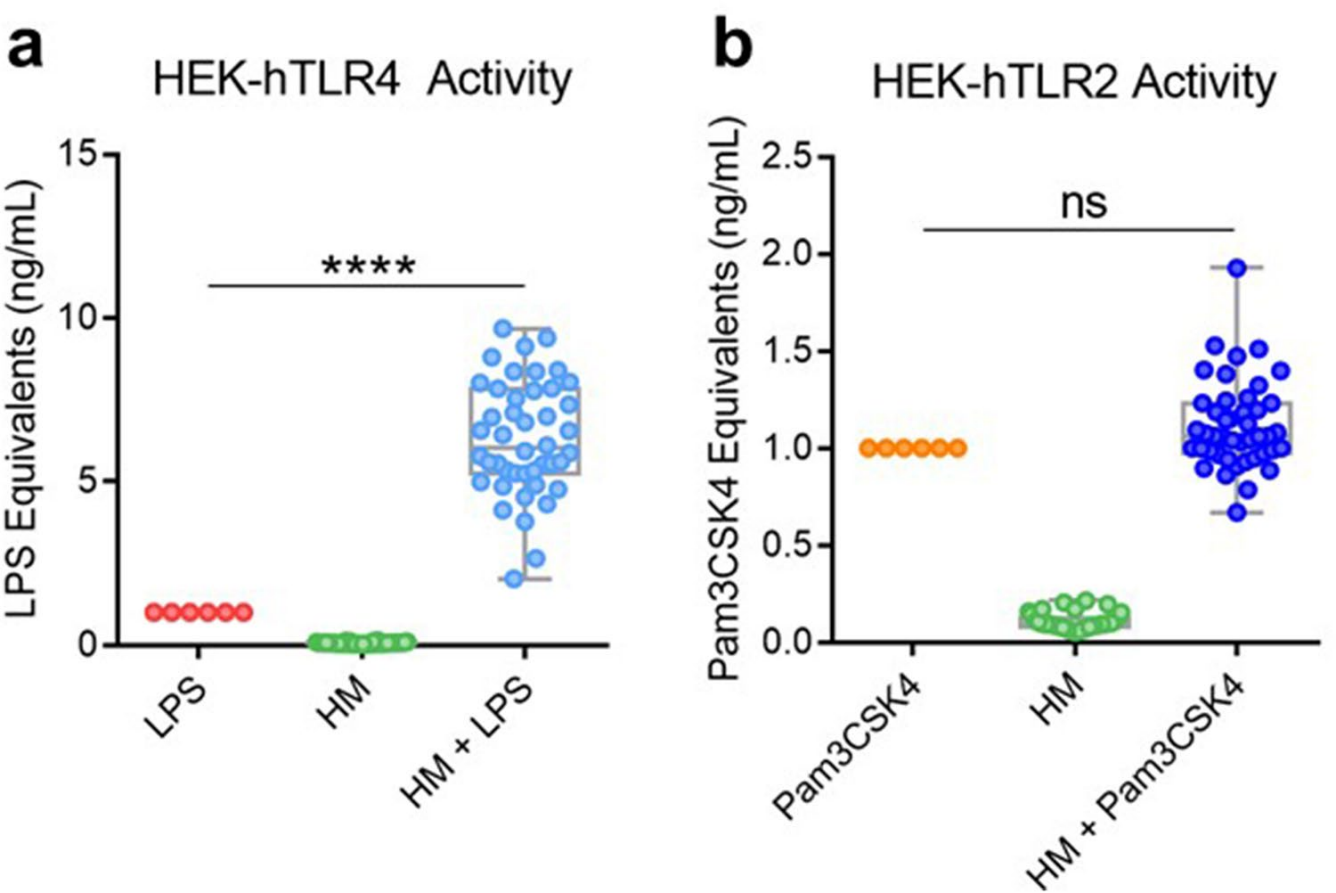
Human milk synergizes with LPS to induce TLR4 activation in HEK-hTLR4 cells. (**a**) HEK-hTLR4 cells were incubated overnight with 333 ng/mL LPS (*n* = 6), 10% HM (*n* = 44), or 10% HM + 333 ng/mL LPS (*n* = 44) and TLR4-induced NF-κB activation was assessed using a SEAP assay. Each point in the HM and HM + LPS conditions represents a HM sample from a single individual. Data are represented as LPS equivalents and normalized to LPS only response. (**b**) HEK-hTLR2 cells were incubated overnight with 12.5 µg/mL Pam3CSK4 (*n* = 6), 10% HM (*n* = 44), or 10% HM + 12.5 µg/mL Pam3CSK4 (*n* = 44) and TLR2-induced NF-κB activation was assessed using a SEAP assay. Each point in the HM and HM + Pam3CSK4 conditions represents a HM sample from a single individual. Data are represented as Pam3CSK4 equivalents and normalized to Pam3CSK4 only response. Box indicates interquartile range and median. Whiskers extend from minimum to maximum. ****P < 0.0001. NS, not significant. LPS, lipopolysaccharide. HM, human milk. Pam3CSK4, Pam3CysSerLys4.

HM is encountered in the neonatal gut in the presence of microbes possessing LPS. To investigate the effects of HM on LPS-induced TLR4 signaling, we treated a HEK-hTLR4 cell line with both HM and LPS. Upon treatment, we observed a significant increase in signal from the HEK-hTLR4 line (Fig. 1a). This increase was beyond an additive effect of the individual LPS and HM conditions alone, indicating that HM enhances LPS TLR4 agonist activity under these circumstances. In comparison, when using HEK-hTLR2 cell line overexpressing a TLR2 gene construct instead of TLR4, no such enhancement in signal was observed when treated with TLR2 agonist Pam3CSK4 in the presence of HM. (Fig. 1b). This demonstrates that this phenomenon of the ability of HM to enhance NF-κB activation is specific to the TLR4 receptor and/or its ligands.

### Human milk enhances LPS-induced expression of pro-inflammatory cytokines as well as transcription and translation regulators in intestinal epithelial cells

**Figure 2:**
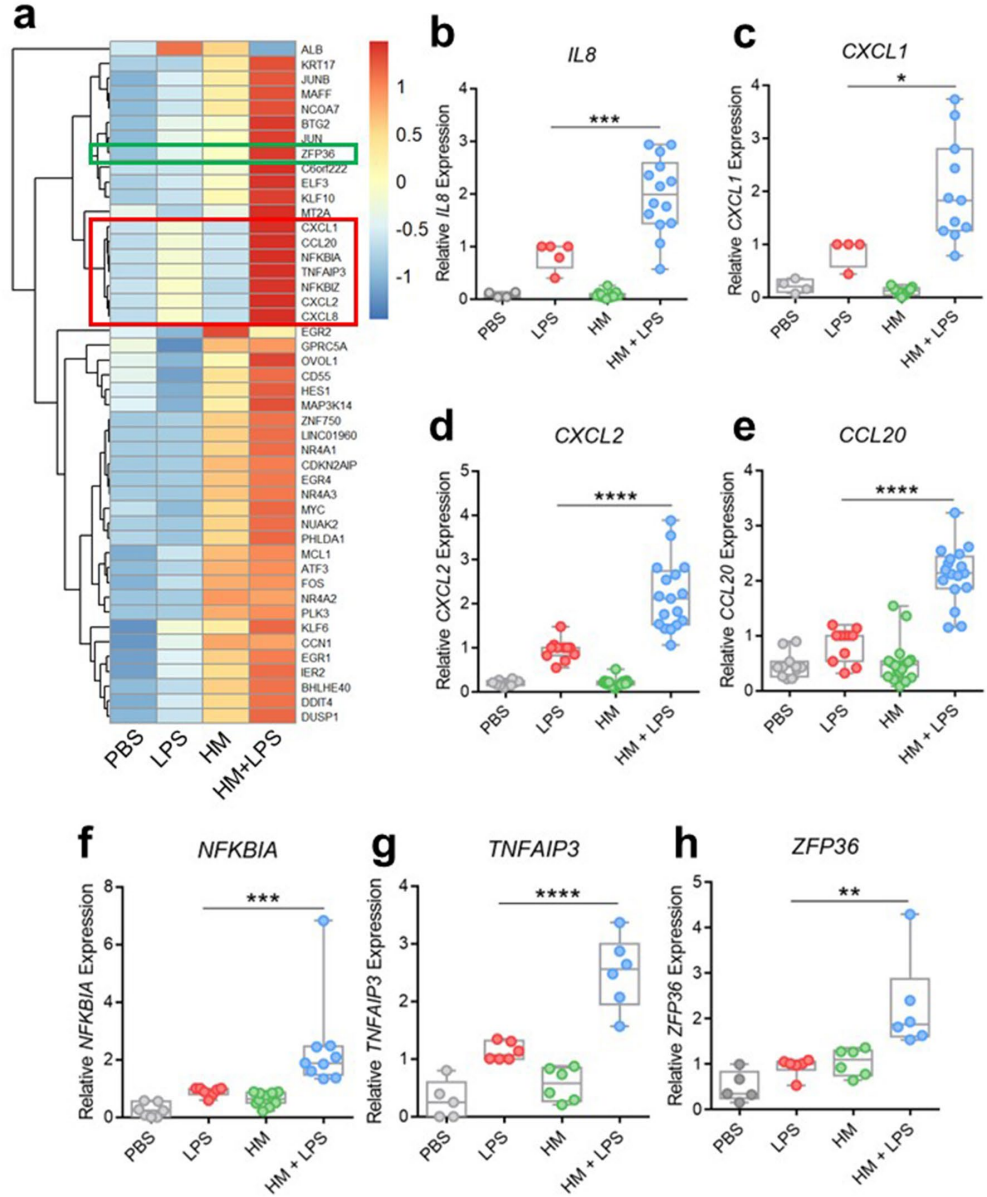
Human milk enhances LPS-induced Caco-2 gene expression of cytokines and transcription and translation regulators. (**a**) RNAseq z-score heat map of genes differentially expressed between Caco-2 cells treated for one hour with 1 µg/mL LPS versus 10% HM (one farming lifestyle sample) + 1 µg/mL LPS. Red box indicates genes that share similar expression patterns. Green box indicates additional gene of interest. Expression of (**b**) *IL8* (PBS *n* = 4, LPS *n* = 5, HM *n* = 15, HM + LPS *n* = 14), (**c**) *CXCL2* (PBS *n* = 10, LPS *n* = 11, HM *n* = 19, HM + LPS *n* = 17), (**d**) *CCL20* (PBS *n* = 11, LPS *n* = 11, HM *n* = 19, HM + LPS *n* = 17), (**e**) *CXCL1* (PBS *n* = 4, LPS *n* = 4, HM *n* = 13, HM + LPS *n* = 11), (**f**) *NFKBIA* (PBS *n* = 7, LPS *n* = 7, HM *n* = 11, HM + LPS *n* = 9), (**g**) *TNFAIP3* (PBS *n* = 5, LPS *n* = 6, HM *n* = 6, HM + LPS *n* = 6), and (**h**) *ZFP36* (PBS *n* = 5, LPS *n* = 6, HM *n* = 6, HM + LPS *n* = 6) by Caco-2 cells treated with PBS, LPS, HM, or HM + LPS was quantified using qRT-PCR. Each point in the HM and HM + LPS conditions represents a HM sample from a single individual. Box indicates interquartile range and median. Whiskers extend from minimum to maximum. *P < 0.05, **P < 0.01, ***P < 0.001, ****P < 0.0001. PBS, phosphate buffered saline. LPS, lipopolysaccharide. HM, human milk.

To further investigate the effect of HM on TLR4 activation by LPS in a more physiological model, we performed similar experiments using the Caco-2 intestinal epithelial cell line. We treated Caco-2 cells with PBS, LPS, HM, or HM + LPS and analyzed subsequent gene expression. One farming lifestyle HM sample was utilized for RNAseq analysis. To determine the effects of HM on LPS-induced gene expression, we compared the RNAseq data between the LPS and HM + LPS treatment conditions, which revealed 46 differentially expressed genes between groups (Fig. 2a, Supplemental Table 2). Interestingly, the HM + LPS treatment group had synergistically enhanced gene expression of a cluster of genes in comparison to the LPS or HM treatment group alone (Fig. 2a). The most notable genes differentially expressed in this pattern include chemokines *CXCL1*, *CXCL2*, *CXCL8* (*IL8*), *CCL20*, and NF-κB inhibitors *NFKBIA* and *TNFAIP3* (A20). The expression of *ZFP36,* a regulator of cytokine translation, was also enhanced in the HM + LPS treatment condition, although it was also induced by HM alone.

We sought to confirm the RNAseq results and investigate the effects of individual HM samples on gene expression using qRT-PCR. Similar to our RNAseq data, HM significantly enhanced LPS-induced *IL8*, *CXCL2*, *CCL20*, *CXCL1*, *NFKBIA*, *TNFAIP3*, and *ZFP36* expression compared to LPS alone, whereas HM alone had minimal effect (Fig. 2b-h).

### Human milk from farming or urban lifestyle mothers exhibit similar enhancement of LPS-induced TLR4 activation and Caco-2 *IL8* expression

**Figure 3:**
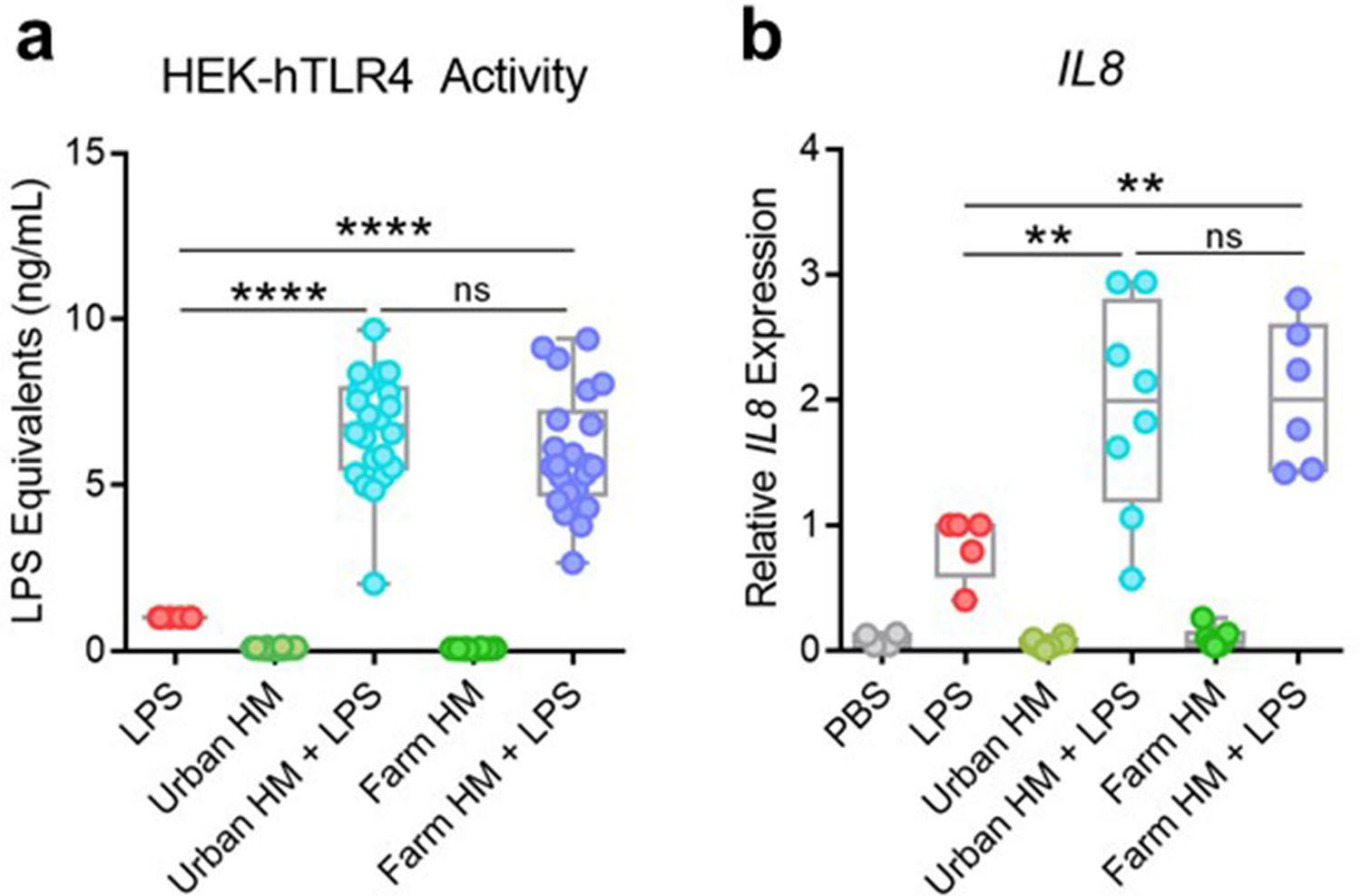
Farming and urban mothers’ human milk enhance LPS-induced TLR4 activation and Caco-2 *IL8* expression comparably. (**a**) HEK-hTLR4 cells were incubated overnight with 333 ng/mL LPS (*n* = 6), 10% urban HM (*n* = 22), 10% urban HM + 333 ng/mL LPS (*n* = 22), 10% farm HM (*n* = 22), or 10% farm HM + 333 ng/mL LPS (*n* = 22) and TLR4-induced NF-κB activation was assessed using a SEAP assay. Data are represented as LPS equivalents and normalized to LPS only response. (**b**) *IL8* gene expression of Caco-2 cells treated with PBS (*n* = 4), LPS (*n* = 5), urban HM (*n* = 8), urban HM + LPS (*n* = 8), farm HM (*n* = 7), or farm HM + LPS (*n* = 6) for one hour quantified using qRT-PCR. Each point in the HM and HM + LPS conditions represents a HM sample from a different individual. Box indicates interquartile range and median. Whiskers extend from minimum to maximum. **P < 0.01, ****P < 0.0001. PBS, phosphate buffered saline. LPS, lipopolysaccharide. HM, human milk.

Our previous studies demonstrate that HM composition varies between farming and urban lifestyle mothers^11^. To determine whether differences in HM composition from these communities affect the synergy between HM and LPS in TLR4 activation and pro-inflammatory cytokine expression, we reanalyzed our data based on the origin of each HM sample. Our data revealed that LPS-induced TLR4 activation and Caco-2 *IL8* expression by HM samples was comparable when using samples from farming and urban lifestyle mothers (Fig. 3a,b).

### Human serum albumin mediates the enhancement of LPS-induced TLR4 activation by human milk

**Figure 4:**
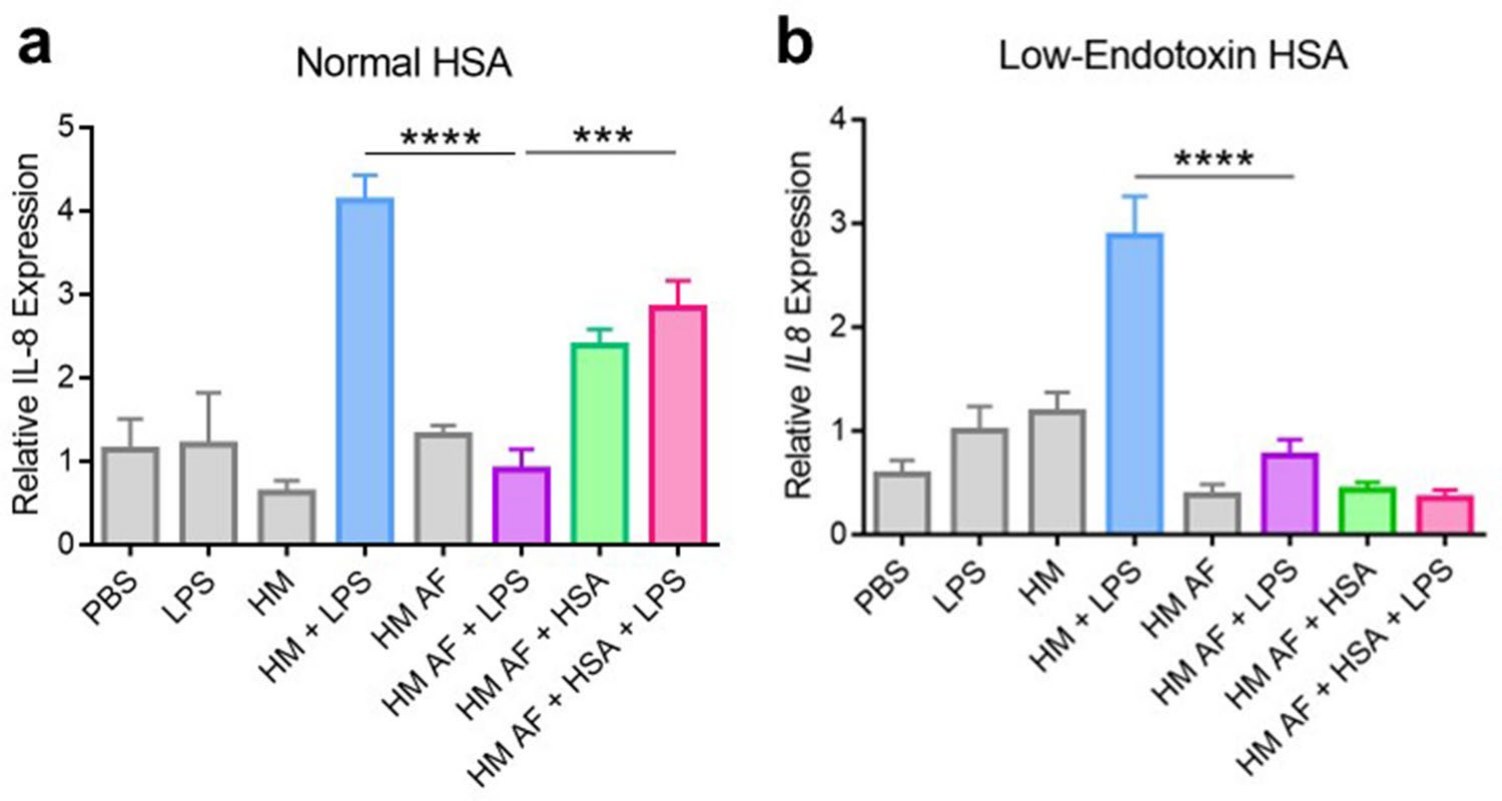
Human serum albumin within human milk contributes to human milk's synergistic effect with LPS on *IL8* expression. Caco-2 *IL8* gene expression in response to one hour treatment with the listed conditions was quantified using qRT-PCR. Treatments containing HSA were either treated with (**a**) 10 µg/mL Fraction V HSA (referred to as normal HSA) or (**b**) 10 µg/mL endotoxin-free HSA. Panels a and b were derived from two independent experiments with two different HM samples. Each panel is a representative trial of the results obtained from three independent experiments. Box indicates interquartile range and median. Whiskers extend from minimum to maximum. **, *P* < 0.01, ***P < 0.001, ****P < 0.0001. PBS, phosphate buffered saline. LPS, lipopolysaccharide. HM, human milk. HM AF, albumin-free human milk. HSA, human serum albumin.

We next focused on identifying the factor(s) in HM that enhance TLR4-induced NF-κB activation during co-treatment with both HM and LPS. Using FPLC, we conducted a series of biochemical separations to enrich the enhancement factor in HM. Using anion exchange chromatography at pH 8.2 and size exclusion chromatography, we isolated several HM fractions which were then used to treat HEK-hTLR4 cells to determine the fraction(s) with the greatest TLR4 enhancing activity. The greatest activity was observed in three contiguous fractions which were then pooled and named the high activity fraction (HF) (Supplemental Fig. 1a,b). Using our HEK-TLR4 model, we confirmed the enhancing effects of HF on LPS-induced TLR4 activation (Supplemental Fig. 1c). Protein sequencing of the purified HF confirmed it to be predominantly composed of HSA (Supplemental Table 3). HSA has been previously shown to act as a co-receptor of TLR4 that enhances the ability of LPS to activate TLR4^10^, and here, we demonstrated that it also enhances LPS mediated signaling of TLR4 in the HEK-hTLR4 cell line (Supplemental Fig. 1d). To further confirm that HSA in HM contributes to the synergy between HM and LPS to induce pro-inflammatory cytokine expression in our Caco-2 model, we used antibody-mediated magnetic bead separation to deplete HM of HSA. Caco-2 cells were grown in serum-free medium to remove confounding effects of albumin present in FBS. They were then treated with HM with or without LPS or albumin-free HM with or without LPS. In comparison to LPS alone, HM + LPS had a significant increase in *IL8* gene expression consistent with our previous data (Fig. 4a,b). However, albumin-free HM plus LPS did not demonstrate this effect and had lower *IL8* expression compared to HM + LPS, illustrating that HSA contributes to the synergistic effect of HM and LPS on Caco-2 cell pro-inflammatory cytokine expression.

To further confirm the role of HSA in this process, we added exogenous Fraction V HSA to the albumin-free HM with or without LPS conditions. In comparison to the resultant Caco-2 *IL8* expression following treatment with albumin-free HM with LPS, Caco-2 *IL8* expression was significantly elevated when HSA was added back into that treatment condition, demonstrating a rescue effect (Fig. 4a). However, treatment of Caco-2 cells with albumin-free HM and HSA elevated *IL8* expression similarly to that of the albumin-free HM with LPS and HSA treatment condition, suggesting potential endotoxin contamination in the HSA. The HSA preparation was subsequently determined to contain 0.68 EU/mg of endotoxin. To address this, we performed the same experiment using commercially available low endotoxin HSA (Fig. 4b). Interestingly, we did not observe the rescue effect with low endotoxin HSA. Similarly, addition of low endotoxin HSA alone to the albumin-free HM treatment condition did not elicit an increase in *IL8* expression.

## Discussion

Human milk is a heterogeneous substance that contains a variety of factors that influence infant mucosal immunity. In this study, we have identified HSA as a component that enhances LPS-induced IEC inflammatory responses and associated negative feedback loops. More specifically, using RNAseq, we identified several LPS-induced genes that were upregulated in response to HM. These genes include pro-inflammatory chemokines (*IL8, CXCL1, CXCL2, CCL20)*, and transcription negative regulators of NF-κB *(NFKBIA* and *TNFAIP3),* as well as cytokine translation regulator *ZFP36*. Additionally, with our HEK-hTLR4 reporter model, we observed that LPS-induced TLR4 signaling was enhanced with the addition of HM or HSA. Given these findings, we postulate that these substances may impact pro-inflammatory responses by modulating TLR4 signaling in IECs. Altogether, the potential of HM HSA to enhance TLR4 signaling suggest HM may enhance the neonatal immune system.

Through protein purification and sequencing, we were able to identify HSA as a mediating factor of the synergistic effect observed between HM and LPS on TLR4 signaling in our HEK-hTLR4 reporter model. HSA is the predominant protein present in human plasma and acts as a carrier protein for several molecules, such as fatty acids, metals, hormones, and LPS^12,13^. Because of HSA's significant carrier function, it is difficult to ascertain whether the synergistic TLR4 activating effects seen between HSA and LPS are due to pure HSA or a factor bound to HSA.

In our Caco-2 model, we depleted HM of HSA and assessed the effects of albumin-free HM on LPS-induced *IL8* expression. We found that depletion of HSA from HM fully attenuated the previously observed synergistic effects between HM and LPS. Although this suggests that HM HSA may be responsible for the enhancing effect of HM on TLR4 activation and *IL8* expression, it could also be due to a factor HSA is carrying rather than HSA itself. Reintroduction of normal HSA into the albumin-free HM plus LPS treatment condition restored synergy between HM and LPS, demonstrating a rescue effect; however, when low-endotoxin HSA was used, no rescue effect was observed. We hypothesize that this may be a result of either (1) alteration of HSA structure so that it can no longer exert its enhancing effects or (2) removal of the true synergistic factor bound to HSA during the endotoxin removal process. Prior literature demonstrates that HSA possesses the capacity to deliver LPS to its obligatory co-receptor MD-2, facilitating activation of TLR4^10^, suggesting a potential mechanism by which HSA itself contributes to the synergistic effect between HM and LPS on TLR4 activation and *IL8* expression. Given this, we are more inclined to believe HSA itself is the synergistic factor. However, this does not rule out the possibility that another molecule bound to HSA could be the true effector.

While there is evidence of anti-inflammatory properties of HM, there are also several HM components that are pro-inflammatory^1^. This implies that the effects of HM and its components on infant immunity is multifactorial, with some components promoting gut homeostasis and dampening inflammation while others enhance inflammation, which may aid in mounting appropriate responses to infection. Among those, the levels of LPS in human milk were initially of interest to us. However, as we found that there were negligible amounts of LPS within our HM samples, we focused on the other aspects of HM that could be boosting or antagonizing the activity of LPS in the infant gut. Overall, our study demonstrates that HM synergizes with LPS to activate TLR4 and increase NF-κB activity. In a gut epithelial cell line, HM enhances LPS-induced expression of pro-inflammatory cytokines as well as regulators of NF-κB and cytokine translation. The effect of HM to synergistically enhance LPS-induced *NFKBIA* and *TNFAIP3* expression suggests that, despite an initial upregulation of inflammatory cytokines (in the case of proinflammatory LPS, P-LPS), there is a negative feedback loop causing suppression of NF-κB as well as its associated induction of inflammation in gut epithelial cells. Altogether this implies that HM may enhance the innate immune response to LPS-containing pathogens without causing chronic inflammation. This provides potential mechanistic evidence behind the protective effects of breastfeeding on both respiratory and gastrointestinal infections in infancy^14^.

While there is quite a low biomass of microbiota in human milk^15^, the largest contribution of LPS seen in the infant GI tract is not from human milk but from other sources feeding and seeding the GI tract. Interestingly, not all LPS is inflammatory^16^, and a group of LPS molecules produced by certain Bacteroidetes are known for their anti-inflammatory properties (A-LPS). Thereby, the effects of HM boosting LPS activity could be also anti-inflammatory in the event that LPS activity boosted by HM is actually anti-inflammatory in nature.

One limitation of this study is the utilization of the Caco-2 cell line, which is derived from adult human colorectal adenocarcinoma. It is quite possible that neonatal IECs may respond to HM in a different manner. However, there are currently no commercially available human neonate-derived IEC lines. Fetal IEC lines, such as FHs 74 int and H4, are available, but they do not form polarized monolayers and are therefore morphologically less physiologic in vitro. Although primary human IECs remain an option, the accessibility of neonatal intestinal epithelium biopsies is very limited and cultures are challenging to maintain.

In conclusion, our study has identified a novel HM factor, HSA, which may play a role in enhancing the innate immune function of IECs in breastfed infants.

## Materials and methods

### Isolation of Human Milk Supernatants

HM was obtained from a cohort of traditional farming lifestyle and urban mothers at 1–3 months postpartum and cryopreserved as part of a study that assessed the effect of farming lifestyle on maternal and infant immunity^11^. Traditional farming lifestyle and urban lifestyle mothers in this cohort were recruited from the Old Order Mennonite (OOM) population of western New York and suburban/urban Rochester, NY, respectively. OOM mothers are known to have increased rates and duration of breastfeeding in comparison to their urban lifestyle counterparts^17,18^. Cryopreserved HM samples were thawed and milk fat and cells were eliminated from HM samples using differential centrifugation followed by filtration through a 0.22 μm filter. Isolated supernatants were utilized in experiments.

### HEK-hTLR4 cell experiments

The human TLR-transfected cell lines HEK-Blue hTLR4 and HEK-Blue hTLR2 were purchased from Invivogen (San Diego, CA) and maintained in an incubator at 37 °C in 5% CO_2_. The morphology of these cells is epithelial. Cells were cultured in Dulbecco’s Modified Eagle Medium (DMEM, Gibco, Grand Island, NY) supplemented with 10% fetal bovine serum (FBS) (Corning, Woodland, CA), 1X penicillin/streptomycin (Gibco, Grand Island, NY), 100 ug/mL Normocin (InvivoGen, San Diego, CA), and 2 mM L-glutamine (Gibco, Grand Island, NY), as well as the proper selection of reagents as per the manufacturer’s instructions. For cell line assays, HEK-Blue pre-defined detection media (Invivogen, San Diego, CA) absent of FBS was re-suspended as per the manufacturer’s instructions, protected from light, stored at 4 °C, and used within 1 week of reconstitution. Assay standards of LPS (Sigma-Aldrich, St. Louis, MO) were thawed, vortexed, and serially diluted 1:3 in phosphate buffered saline (PBS) (Gibco, Grand Island, NY) from a 250 ug/mL stock. Cells were treated with 12.5 µg/mL Pam3-Cys-Ser-Lys 4 (Pam3CSK4) (InvivoGen, San Diego, CA) or 333 ng/mL of LPS spike-in along with 10% HM supernatants or high activity fraction (HF) as appropriate. Cells at 50–80% confluence were then detached, counted, and re-suspended in warm HEK-Blue detection media and seeded in 96-well tissue culture treated microplates as per the manufacturer’s instruction. Plates were then incubated at 37 °C in 5% CO2 for 16–18 h and NF-κB-inducible secreted embryonic alkaline phosphatase enzyme (SEAP) was read on a BioTek Epoch spectrophotometer (Winooski, VT) at optical density 620 (OD 620). Data was normalized to LPS.

### Fast protein liquid chromatography (FPLC)

Prior to FPLC, prepared HM samples were casein depleted. Pre-cleared HM pH was lowered to 4.5 using 2 M HCl. Precipitated casein was then removed by centrifugation at 4400 rpm for 30 min at room temperature. Supernatants were isolated and pH was returned to 6.6. These supernatants were then fractionated via anion exchange chromatography using a HiTrap Q FF 5 × 5 mL column (GE Healthcare, Uppsala, Sweden) with a buffer gradient starting with 20 mM Tris 35 mM NaCl to 20 mM Tris 1 M NaCl, pH 8.2. Active fractions where further purified via size exclusion chromatography using a Superdex 200 10/300 GL column (GE Healthcare, Uppsala, Sweden). Effluent protein concentration was monitored by absorbance at 280 nm. Fractions were tested in HEK-hTLR4 assays, detailed above, to identify fractions containing enhancing effect on LPS-signaling.

### Mass spectrometry

The high activity HM fraction was run in a 4–12% sodium dodecyl-sulfate polyacrylamide gel electrophoresis (SDS-PAGE) gel to separate proteins. The region of interest was excised and dehydrated with acetonitrile. Peptides were extracted the next day by addition of 50% acetonitrile, 0.1% trifluoroacetic acid (TFA), then dried down in a CentriVap concentrator (Labconco, Kansas City, MO). A Fusion Lumos Tribrid mass spectrometer (Thermo Fisher) was used to analyze the peptides. Raw data was searched using the SEQUEST search engine within the Proteome Discoverer software platform, version 2.4 (Thermo Fisher), using the SwissProt *Homo sapiens* database. Percolator was used as the false discovery rate (FDR) calculator, filtering out peptides which had a q-value greater than 0.01.

### Human serum albumin depletion

HSA was depleted from HM supernatants using PureProteome Albumin Magnetic Beads (Millipore, Burlington, MA) according to the manufacturer’s protocol. Isolated albumin-free (AF) HM supernatants were then concentrated using Amicon Ultra 0.5 mL 3 K centrifugal filters (Millipore, Burlington, MA) and centrifuged down at 14,000xg for 5 min. Concentrated samples were then sterile filtered through a 0.22 μm filter.

### Endotoxin measurement

Endotoxin content of Fraction V HSA (Sigma-Aldrich, St. Louis, MO) was determined from a 50 μg/mL solution of HSA using Pierce Chromogenic Endotoxin Quant Kit (Thermo Scientific, Rockford, IL) using kit instructions.

### Caco-2 cell experiments

Caco-2 cells were cultivated in DMEM, 10% FBS, 4 mM L-glutamine, 1X penicillin/streptomycin or DMEM, 1X insulin-transferrin-selenium (Gibco, Grand Island, NY), 4 mM L-glutamine, 1X penicillin/streptomycin for HSA depletion experiments at 37 °C 5% CO_2_. 1 × 10^5^ cells were seeded in the upper chamber of 24-well plate transwells on a polycarbonate membrane (0.4 μm diameter pores) (Corning Costar, Corning, NY). Cells were cultured for 7–21 days until the trans-epithelium electrical resistance (TEER), as measured using a chopstick electrode ohmmeter, reached at least 300 Ω × cm^2^. 10% PBS, 1 μg/mL LPS, 10% HM supernatants, 10% HM supernatants plus 1 μg/mL LPS, 10% albumin-free (AF) HM supernatants, 10% AF HM supernatants plus 1 μg/mL LPS, 10% AF HM supernatants plus 10 μg/mL Fraction V HSA (referred to as "normal HSA") (Sigma-Aldrich, St. Louis, MO) or low-endotoxin HSA (Sigma-Aldrich, St. Louis, MO), or 10% AF HM supernatants plus 1 μg/mL LPS plus 10 μg/mL Fraction V HSA (Sigma-Aldrich, St. Louis, MO) or low-endotoxin HSA (Sigma-Aldrich, St. Louis, MO) were added in the upper chamber and incubated for one hour at 37 °C 5% CO_2_. Cells were lysed using RLT Lysis Buffer and RNA was extracted using Qiagen RNeasy Plus Mini Kit (Qiagen, Germantown, MD). For quantitative real-time (qRT) PCR gene expression studies, RNA was reverse transcribed into cDNA using the iScript cDNA Synthesis Kit (BioRad, Hercules, CA).

### RNAseq and analysis

RNA concentration was determined with the NanopDrop 1000 spectrophotometer (NanoDrop, Wilmington, DE) and RNA quality assessed with the Agilent Bioanalyzer 2100 (Agilent, Santa Clara, CA). 1 ng of total RNA was pre-amplified with the SMARTer Ultra Low Input kit v4 (Clontech, Mountain View, CA) per manufacturer’s recommendations. 150 pg of cDNA was used to generate Illumina compatible sequencing libraries with the NexteraXT library preparation kit (Illumina, San Diego, CA) per manufacturer’s protocols. The amplified libraries were hybridized to the Illumina flow cell and sequenced using the NextSeq 550 sequencer (Illumina, San Diego, CA). Single end reads of 75 nucleotides (nt) were generated for each sample.

Raw reads generated from the Illumina basecalls were demultiplexed using bcl2fastq version 2.19.0. Quality filtering and adapter removal are performed using FastP version 0.20.1. Processed/cleaned reads were then mapped to the human reference genome (GRCh38.p13) using STAR_2.7.6a. Gene level read quantification was derived using the subread-2.0.1 package (featureCounts) with a GTF annotation file (Genocode-31). Differential expression analysis was performed using DESeq2-1.28.1 with a P-value threshold of 0.05 within R version 4.0.2 (https://www.R-project.org/). Z-score heatmaps of normalized counts were generated using the pheatmap package.

### Quantitative real-time PCR (qRT-PCR)

Expression of *IL8, CXCL1, CXCL2, CCL20, TNFAIP3* (*A20*), *NFKBIA,* and *ZFP36* was compared to reference gene *B2M* (*β2 microglobulin*) using a TaqMan probe-based qPCR gene expression assay (Applied Biosystems, Warrington, United Kingdom) performed on a BioRad CFX96 Real-Time system. The assay was carried out using the manufacturer’s TaqMan qRT-PCR protocol using pre-made primer/probe sets (IDT, Morrisville, NC) for each gene of interest (Supplemental Table 1). Assays were conducted in technical triplicate. The relative expression of genes of interest was calculated using the 2^*−ΔΔCT*^ method and analyzed using the CFX Maestro software (BioRad). Data was normalized to the LPS control across each plate of biological replicates.

### Statistics

GraphPad Prism was used to graph and perform a one-way analysis of variance (ANOVA) with a post-hoc Tukey test to statistically analyze all data. Patterns were deemed synergistic if the average relative gene expression of Caco-2 cells treated with both HM and LPS was greater than the sum of the average relative gene expression of Caco-2 cells treated with HM or LPS alone.

### Ethics approval and consent to participate

Samples collected were a part of a previous study from our group that assessed the effect of farming lifestyle on maternal and infant immunity^11^. All participants were recruited after written informed consent. The study protocol was approved by the University of Rochester Medical Center Research Subjects Review Board and all experiments were performed in accordance with relevant guidelines and regulations.

### Supplementary Information

Supplementary Information.

## Data Availability

The datasets generated during and/or analyzed during the current study are available from the corresponding author on reasonable request.
