# Supplementary material for: Human milk affects TLR4 activation and LPS-induced inflammatory cytokine expression in Caco-2 intestinal epithelial cells
Source: Sci Rep. 2024 Jun 11;14:13448. doi: 10.1038/s41598-024-64000-z (PMC11167050; doi:10.1038/s41598-024-64000-z)
Supplement: Supplementary file 1 — Supplementary Information. [file 41598_2024_64000_MOESM1_ESM.pdf]

Target Name	Assay ID	SequenceName	Sequence
IL8	Hs.PT.58.39926886.g	PrimeTime Primer 1	CTTCACACAGAGCTGCAGAA
		PrimeTime Primer 2	GAGACAGCAGAGCACACAAG
		PrimeTime Probe	/56-FAM/AGGACAAGA/ZEN/GCCAGGAAGAAACCAC/3IABkFQ/
B2M	Hs.PT.58v.18759587	PrimeTime Primer 1	ACCTCCATGATGCTGCTTAC
		PrimeTime Primer 2	GGACTGGTCTTTCTATCTCTTGT
		PrimeTime Probe	/5Cy5/CCTGCCGTGTGAACCATGTGACT/3IAbRQSp/
CXCL1	Hs.PT.58.39039397	PrimeTime Primer 1	TCTCTCTTTCCTCTTCTGTTCCTA
		PrimeTime Primer 2	CATCCCCCATAGTTAAGAAAATCATC
		PrimeTime Probe	/56-FAM/AAGCTCACT/ZEN/GGTGGCTGTTCCT/3IABkFQ/
ZFP36	Hs.PT.58.3309682.g	PrimeTime Primer 1	GAGACCACAGTGCAAGAGAC
		PrimeTime Primer 2	CCCATCTTCAATCGCATCTCT
		PrimeTime Probe	/56-FAM/TTCTGAGTG/ZEN/ACAAAGTGACTGCCCG/3IABkFQ/
NFKBIA	Hs.PT.58.15498666.g	PrimeTime Primer 1	CATTGACATCAGCACCCAAG
		PrimeTime Primer 2	TCCTGAAGGCTACCAACTACA
		PrimeTime Probe	/56-FAM/ACCAAAAGC/ZEN/TCCACGATGCCCA/3IABkFQ/
TNFAIP3	Hs.PT.58.1824217	PrimeTime Primer 1	TCCTGCCATTTCTTGTACTCAT
		PrimeTime Primer 2	TGATAGAAATCCCCGTCCAAG
		PrimeTime Probe	/56-FAM/AGCTTCATC/ZEN/CAACTTTGCGGCATTG/3IABkFQ/
CCL20	Hs.PT.58.19600309	PrimeTime Primer 1	TTAGGATGAAGAATACGGTCTGTG
		PrimeTime Primer 2	CCATGTGCTGTACCAAGAGT
		PrimeTime Probe	/56-FAM/TGTCAGTGC/ZEN/TGCTACTCCACCTCT/3IABkFQ/

Supplemental Table 1: Primer and probe sequences for TaqMan qRT-PCR assays.

	BaseMean	log2FoldChange	stat	pvalue	padj
NR4A1	3834.642	1.153	12.049	1.97E-33	2.93E-29
FOS	9894.297	0.843	11.126	9.34E-29	6.93E-25
TNFAIP3	2901.013	1.009	9.245	2.36E-20	1.17E-16
NFKBIA	3755.96	0.917	9.068	1.21E-19	4.48E-16
DUSP1	4855.943	0.723	8.856	8.28E-19	2.46E-15
CXCL2	1469.374	1.233	8.264	1.41E-16	2.99E-13
KLF10	9027.747	0.617	8.266	1.38E-16	2.99E-13
JUNB	3446.295	0.763	7.952	1.83E-15	3.40E-12
NR4A3	318.197	0.675	7.884	3.18E-15	5.25E-12
NFKBIZ	4004.896	0.818	7.661	1.84E-14	2.74E-11
CCL20	743.406	1.04	7.434	1.06E-13	1.43E-10
ZFP36	2678.775	0.693	6.668	2.59E-11	3.20E-08
BTG2	4143.496	0.525	6.17	6.82E-10	7.56E-07
EGR1	4252.213	0.578	6.163	7.13E-10	7.56E-07
LINC0196(	372.864	0.552	6.018	1.77E-09	1.75E-06
PLK3	1096.681	0.573	5.685	1.31E-08	1.21E-05
JUN	1876.14	0.564	5.587	2.30E-08	2.01E-05
BHLHE40	1646.539	0.531	5.438	5.38E-08	4.44E-05
KRT17	1208.273	0.549	5.308	1.11E-07	8.66E-05
HES1	2490.329	0.465	5.093	3.52E-07	0.000261
NCOA7	7259.956	0.4	4.937	7.93E-07	0.00056
ATF3	2195.82	0.469	4.911	9.05E-07	0.00061
MAFF	1225.939	0.508	4.809	1.51E-06	0.000977
ELF3	7201.759	0.406	4.795	1.63E-06	0.001005
OVOL1	3346.526	0.395	4.578	4.7E-06	0.002791
ALB	9992.156	-0.368	-4.532	5.84E-06	0.003333
MCL1	6629.92	0.342	4.504	6.67E-06	0.003665
CCN1	7690.089	0.379	4.492	7.04E-06	0.003732
DDIT4	2851.139	0.438	4.389	1.14E-05	0.005831
C6orf222	627.029	0.499	4.335	1.45E-05	0.007195
IER2	2895.364	0.391	4.25	2.14E-05	0.010246
CD55	4302.396	0.338	4.212	2.54E-05	0.011757
EGR4	280.796	0.41	4.175	2.98E-05	0.013415
NUAK2	4846.475	0.336	4.11	3.96E-05	0.016779
MAP3K14	539.343	0.423	4.116	3.86E-05	0.016779
CDKN2AIP	2750.471	0.381	4.074	4.62E-05	0.019038
PHLDA1	2598.485	0.355	3.985	6.74E-05	0.026321
ZNF750	825.472	0.423	3.986	6.71E-05	0.026321
GPRC5A	10778.837	0.284	3.922	8.8E-05	0.033463
EGR2	378.507	0.326	3.914	9.08E-05	0.033684
KLF6	10659.669	0.285	3.894	9.87E-05	0.035729
MT2A	3558.31	0.373	3.876	0.000106	0.037519
NR4A2	145.178	0.261	3.843	0.000122	0.040968
CXCL8	279.188	0.625	3.846	0.00012	0.040968
CXCL1	1151.309	0.545	3.822	0.000132	0.043633
MYC	6360.31	0.33	3.787	0.000152	0.049181

Supplemental Table 2: Differentially expressed genes between human milk plus LPS and LPS only Caco-2 treatment conditions.

Accession	Description	MW [kDa]	# Unique Peptides	# Peptides	# PSMs
P02768	Serum albumin OS=Homo sapiens (Human) OX=9606 GN=ALB PE=1 SV=2	69.3	76	76	936
P01024	Complement C3 OS=Homo sapiens (Human) OX=9606 GN=C3 PE=1 SV=2	187	21	21	23
P07498	Kappa-casein OS=Homo sapiens (Human) OX=9606 GN=CSN3 PE=1 SV=3	20.3	7	7	13
Q08380	Galectin-3-binding protein OS=Homo sapiens (Human) OX=9606 GN=LGALS3BP PE=1 SV=1	65.3	9	9	10
P13796	Plastin-2 OS=Homo sapiens (Human) OX=9606 GN=LCP1 PE=1 SV=6	70.2	6	6	6
P01876	Immunoglobulin heavy constant alpha 1 OS=Homo sapiens (Human) OX=9606 GN=IGHA1 PE=1 SV=2	37.6	5	5	5
P05814	Beta-casein OS=Homo sapiens (Human) OX=9606 GN=CSN2 PE=1 SV=4	25.4	3	3	3
P47710	Alpha-S1-casein OS=Homo sapiens (Human) OX=9606 GN=CSN1S1 PE=1 SV=1	21.7	2	2	2
Q66K66	Transmembrane protein 198 OS=Homo sapiens (Human) OX=9606 GN=TMEM198 PE=1 SV=1	39.4	1	1	2

Supplemental Table 3: Proteins from high activity fraction identified by mass spectrometry. MW, molecular weight. PSM, peptide-spectrum match.

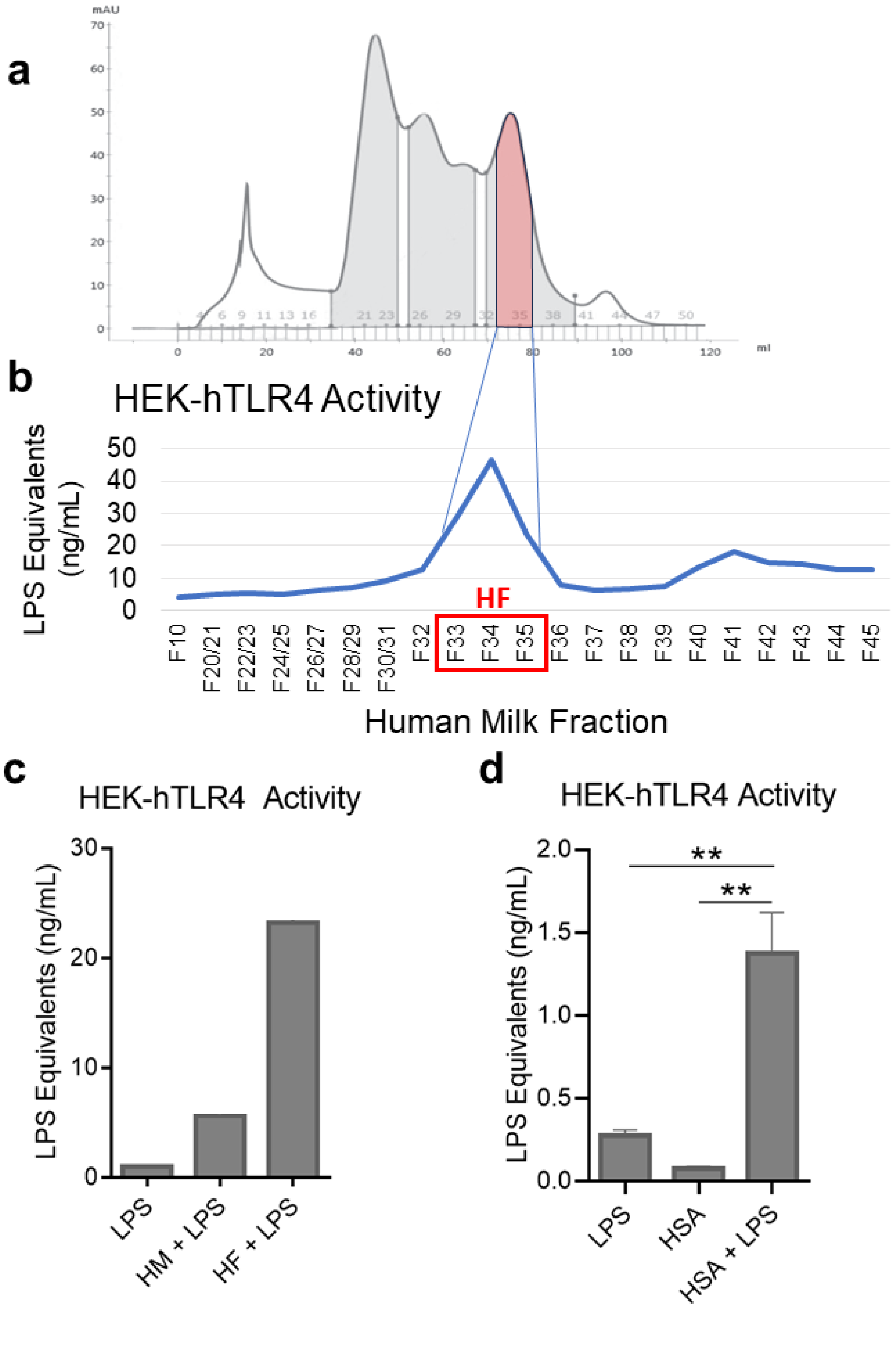
a
mAU
ml
b
HEK-hTLR4 Activity
LPS Equivalents (ng/mL)
HF
F10
F20/21
F22/23
F24/25
F26/27
F28/29
F30/31
F32
F33
F34
F35
F36
F37
F38
F39
F40
F41
F42
F43
F44
F45
Human Milk Fraction
c
HEK-hTLR4 Activity
LPS Equivalents (ng/mL)
LPS
HM + LPS
HF + LPS
d
HEK-hTLR4 Activity
LPS Equivalents (ng/mL)
**
**
LPS
HSA
HSA + LPS

Supplemental Figure 1: Anion exchange chromatographic purification of high activity fraction (HF) from human milk. Defatted human milk was further defatted and cleared by 0.45 micron filtration and applied to a HiQ anion exchange FPLC column (Pharmacia) at 0.5 ml/min. Column was washed with wash buffer (20mM Tris, pH 8.0; 35 mM NaCl) and bound protein eluted with a gradient of 0-500 mM NaCl in starting buffer. Effluent was monitored using a UV-detector at 280 nm and fractions collected as indicated in (a). TLR activity was measured in collected fractions after overnight incubation of HEK-hTLR4 cells with 10% human milk fractions + 333ng/mL LPS, which is shown in (b). High activity fractions 33-35 are highlighted in (a) and (b) in red and were pooled and desalted using a membrane concentrator and designated as the final high activity fraction (HF). TLR4 activity in response to overnight stimulation of HEK-hTLR4 cells with (b) 333ng/mL LPS (*n*=1), 10% HM + 333ng/mL LPS (*n*=2), and 10% HF + 333ng/mL LPS (*n*=2), and (c) 333ng/mL LPS (*n*=3), 10μg/mL normal HSA (*n*=3), and 10μg/mL normal HSA + 333ng/mL LPS (*n*=3). Data are represented as LPS equivalents and normalized to LPS only response. HF, high activity fraction. LPS, lipopolysaccharide. HM, human milk. HSA, human serum albumin.
